# Spectrophotometric determination of ammonia levels in tobacco fillers of and sidestream smoke from different cigarette brands in Japan

**DOI:** 10.1186/s12199-018-0704-5

**Published:** 2018-04-27

**Authors:** Yohei Inaba, Shigehisa Uchiyama, Naoki Kunugita

**Affiliations:** Department of Environmental Health, National Institute of Public Health, 2-3-6 Minami, Wako, Saitama, 351-0197 Japan

**Keywords:** Ammonia, Tobacco filler, Sidestream smoke, Spectrophotometer, Salicylate-chlorine reaction

## Abstract

**Background:**

The ammonia contained in tobacco fillers and mainstream and sidestream cigarette smoke accelerates nicotine dependence in cigarette smokers. Ammonia has been included in the non-exhaustive priority list of 39 tobacco components and emissions of cigarette published by the World Health Organization (WHO) Study Group on Tobacco Product Regulation. The development of a simple ammonia detection method will contribute to the establishment of tobacco product regulation under tobacco control policies and allow surveys to be conducted, even by laboratories with small research budgets.

**Methods:**

We developed a simple colorimetric method based on the salicylate-chlorine reaction and absorption spectrometry with two reagents (sodium nitroprusside and sodium dichloroisocyanurate). To compare this method to conventional ion chromatography, we analyzed the ammonia levels in tobacco fillers extracted from 35 Japanese commercially marketed cigarette brands manufactured by four tobacco companies (Japan Tobacco (JT) Inc., British American Tobacco (BAT), Philip Morris Japan, and Natural American Spirit). We also analyzed the ammonia levels in the sidestream smoke from cigarettes of the brands that were found to contain high or low tobacco filler ammonia levels.

**Results:**

The ammonia levels in the reference cigarette (3R4F) measured by our method and ion chromatography were similar and comparable to previously reported levels. The ammonia levels in tobacco fillers extracted from 35 cigarette brands ranged from 0.25 to 1.58 mg/g. The mean ammonia level of JT cigarette brands was significantly higher (0.83 ± 0.28 mg/g) than that of Natural American Spirit cigarette brands (0.30 ± 0.08 mg/g) and lower than those in the other two cigarette brands (1.11 ± 0.19 mg/g for BAT and 1.24 ± 0.15 mg/g for Philip Morris) (*p* < 0.001 by Bonferroni test). The ammonia levels in the sidestream smoke of CABIN, Marlboro Black Menthol, American Spirit Light, and Seven Stars were 5.89 ± 0.28, 5.23 ± 0.12, 6.92 ± 0.56, and 4.14 ± 0.19 mg/cigarette, respectively. The ammonia levels were higher in sidestream smoke than in tobacco filler.

**Conclusions:**

Our simple colorimetric could be used to analyze ammonia in tobacco fillers and sidestream smoke. There were significant differences between the ammonia levels of the 35 commercially marketed cigarette brands in Japan manufactured by four tobacco manufacturers. Over 90% of the ammonia in sidestream smoke was in gaseous phase.

## Background

Ammonia is a chemical additive found in tobacco filler [1–4]. It increases nicotine dependence in cigarette smokers and has been included in the non-exhaustive priority list of 39 tobacco contents and emissions of cigarette by the World Health Organization (WHO) Study Group on Tobacco Product Regulation [1]. The effects of ammonia present in tobacco filler on cigarette smoke, such as increasing its alkalinity, which in turn increases the amount of unprotonated nicotine in cigarette smoke, have been examined extensively [1]. Unprotonated (“free-base”) nicotine is lipophilic and is absorbed more quickly into the bloodstream than its protonated form [1]. The increase in free-base nicotine increases the addictive potential of cigarettes [2]. Thus, the role of added ammonia in increasing the delivery of free-base nicotine to the brain has been suggested as an ammonia technology. A recent study analyzed the levels of nicotine in the blood of participants who smoked cigarettes containing different levels of ammonia in the tobacco filler, and no differences in nicotine levels were found [5]. As a result of this study, ammonia technology is regarded a legacy technology. However, this study did not use products with very low levels of ammonia in their tobacco fillers; therefore, the influence of the amount of ammonia in tobacco filler on nicotine absorption is still unknown. Currently, tobacco industries are developing alternative technologies and approaches to ammonia technology. There are 600 additives besides ammonia contained in tobacco products (including cocoa, caramel color, menthol, and rum and its flavors) [6]. Furthermore, there are 188 additives listed on the homepage of Japan Tobacco Inc. [7]. For instance, menthol allows the deeper inhalation of cigarette smoke since it has local anesthetic qualities. A deeper puff of smoke is achieved, and the dose of nicotine per puff is higher. The sale of flavored tobacco products, such as menthol capsule cigarettes, is not regulated in Japan. Therefore, manufacturers can use additives other than ammonia, such as menthol and cocoa. As the ammonia levels in commercially marketed cigarette brands in Japan have not been disclosed, the ammonia levels of these cigarettes should be investigated.

The ammonia levels in tobacco fillers of foreign cigarette brands have been analyzed [8–10]. Canadian cigarette brands reportedly contain significantly lower levels of ammonia than US cigarette brands [9]. Moreover, in the USA, ammonia levels differ significantly between tobacco manufacturers [10]. Although several countries now monitor the ammonia levels of cigarettes, the Japanese government has yet to set forth monitoring requirements for ammonia in commercially marketed cigarettes. In 2005, Philip Morris USA reported that the ammonia levels of five cigarette brands sold in Japan ranged from 1.06 to 3.28 mg/g [8]. However, the ammonia levels of Japanese cigarette brands have not been reported.

Another important aspect that needs to be investigated is the amount of ammonia transferred from the tobacco filler to cigarette smoke. The ammonia levels in mainstream smoke have not been determined separately from that transferred from tobacco filler as mainstream smoke contains much less ammonia (3.1–29.0 μg/cigarette according to the International Organization for Standardization (ISO)) [8] than tobacco filler. The US Surgeon General [11] reported that the ammonia levels of sidestream smoke are significantly higher, resulting in a more alkaline pH [12]. Additionally, due to its corrosive and exothermic nature, ammonia can cause immediate injury to the mucosa of the eyes, skin, oral cavity, and respiratory tract. Monitoring hazardous chemicals in tobacco smoke is essential for the regulation of tobacco products. The analysis of toxicants in mainstream smoke is also required according to articles 9 and 10 of the WHO Framework Convention on Tobacco Control (FCTC)—regulation of the contents and disclosures of tobacco products. Accordingly, we hypothesized that the transfer of ammonia from tobacco filler to smoke and differences between ammonia levels of different cigarette brands can be confirmed by analyzing the ammonia levels in sidestream smoke.

The analytical methods for ammonia in tobacco filler are mainly based on ion chromatography. Among those, the methods proposed by the WHO and Cooperation Centre for Scientific Research Relative to Tobacco (CORESTA) are fast and reliable [13, 14]. Ion chromatography is widely used for studying various anions and cations in water and allows simultaneous analysis of cations other than ammonia. Ion chromatography has also been used to analyze ammonia in mainstream cigarette smoke [15]. However, purchasing and maintaining ion chromatography instruments only for analyzing ammonia in tobacco filler and tobacco smoke is relatively expensive. Jansen et al. proposed an analytical method that uses clinical analyzers based on enzyme reactions [16]. In addition, in the water research field, the colorimetric method has been generally followed to determine ammonia levels by using an absorption spectrometer, which is generally available in most analytical laboratories. This method is based on the salicylate-chlorine reaction [17] and has sensitivity and specificity for ammonia analysis of tobacco products. Therefore, we followed this method to analyze the ammonia levels in tobacco filler as well as in sidestream smoke.

The aim of this study was to develop a colorimetric method for determining ammonia levels in tobacco filler and sidestream smoke using an absorption spectrometer and two reagents (sodium nitroprusside and sodium dichloroisocyanurate). The proposed method was compared to ion chromatography, the ammonia levels of different Japanese cigarette brands were measured, and ammonia levels in cigarettes of four tobacco manufacturers in the Japanese market were compared. The proposed method was then used to determine the ammonia levels in sidestream smoke and whether the ammonia detected in sidestream smoke is directly transferred from tobacco filler or is formed through the pyrolysis of the nitrogenous compounds in tobacco filler.

## Methods

### Materials

Standard ammonia (5 mg/L) and multi-cation III solutions were purchased from Wako Pure Chemical Industries (Osaka, Japan). Ultrapure water was produced by a Milli-Q Integral3 System (Merck Millipore, Tokyo, Japan). A Nitrogen-Ammonia Reagent Set, which includes two regents (ammonia cyanurate and ammonia salicylate), was purchased from HACH (Germany).

### Apparatus and analytical condition

The ammonia levels in tobacco filler were analyzed with an ion chromatography system (Dionex ICS-2100 Integrated Reagent-Free, Thermo Fisher Scientific, CA, USA) equipped with a conductivity detector, as reported previously [13, 18]. Ammonia separation was conducted by using a DX IonPac CS16 analytical column (250 mm × 4 mm internal diameter (i.d.)) with a CG16 guard column (50 mm × 4 mm i.d.) and CSRS 300 4 mm RFIC Self-Regenerating Suppressor. A 30-mmol/L solution of methanesulfonic acid was used in the Dionex automatic eluent generator (EGC 500 MSA) at an injection volume of 25 μL and flow rate of 1 mL/min.

### Cigarette samples and storage

Cigarettes of 35 brands, including 11 Japanese and 24 American brands, were purchased from tobacco retail stores in Japan (Table 1). Tobacco filler was removed from each cigarette, which were stored at − 20 °C prior to measurement and conditioned in a CSH-111 stability test chamber (ESPEC Corp., Osaka, Japan) at 22 ± 1 °C and 60 ± 2% humidity for at least 48 h, as specified in ISO 3402 [19].

**Table 1:** Comparison of ammonia in 3R4F tobacco filler by study

Method	Study reference	3R4F ammonia (mg/g)
IC	Watson et al. 2015	1.1 ± 0.14
	Jansen et al. 2014	1.07 ± 0.015
	CORESTA 2015	1.08 ± 0.10
	Current study	1.12 ± 0.12
Colorimetric method	Current study	1.22 ± 0.08

### Sample preparation

Tobacco fillers were extracted according to WHO TobLabNet SOP 7 [13] by using an extraction solution diluted with sulfuric acid at approximately 0.0125 mol/L, and stored in a refrigerator. Tobacco filler sample (0.70 g) was placed in a 250-mL Erlenmeyer flask, and 50 mL of the extraction solution was added. The sample was shaken at 160 rpm for 30 min on a rotary shaker (Double Shaker NR-30, TAITEC, Saitama, Japan), and then kept in dark for 30 min until the supernatant was clear. The supernatant was then filtered through 8-μm ashless quantitative filter paper.

### Sidestream smoke collection and extraction

Sidestream smoke was collected using a single-port piston-type smoking machine (Model LM1/PLUS, Heirich Borgwaldt Hamburg, Germany) according to ISO 3308 [20] under the following conditions: 60 s puff interval, 2 s puff duration, and 35 mL puff volume. The smoke was captured at three positions (the fishtail, Cambridge filter pad (CFP) holder, and impinger stand) at a flow rate of 3 L/min. The impinger solution was prepared in 30 mL of 0.0125-mol/L sulfuric acid. The fishtail and impinger extract solutions were combined and the impinger solution was added to create a volume of 100 mL. Next, the CFP was added to the combined solution and was extracted by shaking for 30 min.

### Colorimetric method

When ammonia reacts with an alkaline mixture of sodium salicylate and sodium dichloroisocyanurate, it generates an emerald green color. The reaction of salicylate-chlorine with ammonia is sensitive, specific, stable, and reproducible [17]. Extract solutions were diluted (1:9, *v*/*v*) with ultrapure water, mixed with ammonia salicylate powder, shaken, and allowed to react for 3 min. Ammonia cyanurate powder was then added, followed by shaking and 15 min of reaction. The absorbance of the final reactant was measured at 660 nm using a spectrophotometer (Auto Sipper Photometer U-2910, Hitachi, Tokyo, Japan).

### Statistical analysis

The ammonia levels in cigarettes of the four manufacturers were compared by using one-way ANOVA, followed by Bonferroni correction for multiple comparisons. *p* < 0.05 was considered to be statistically significant. Statistical analyses were conducted using SPSS version 22.0 for Mac OS X.

## Results and discussion

### Principle of the colorimetric method

The reaction of dilute ammonium sulfate with an alkaline mixture of sodium salicylate and sodium dichloroisocyanurate, which produces a brilliant emerald green color, can be measured by absorption at 660 nm and was proposed by Reardon et al. in 1966 [17]. The colorimetric method based on this reaction has been commercialized for use in water, wastewater, and seawater analyses. We used a commercially available kit for measuring the ammonia levels in tobacco filler.

### Optimal conditions and method validation

**Fig. 1:**
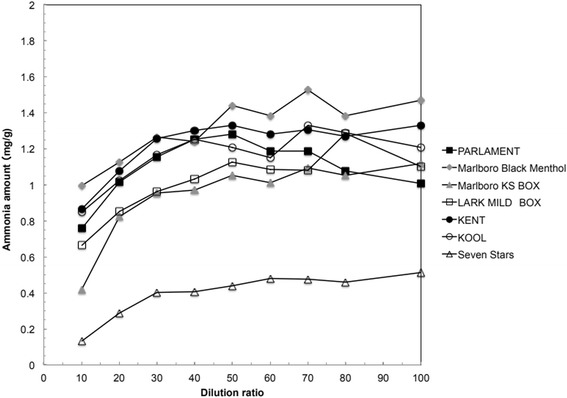
Effects of dilution on ammonia determination with an ammonia test kit

Ammonia extracts were examined to establish proper dilutions (1:5–1:100) for colorimetric analysis (Fig. 1). The optimal dilution was 1:50–1:100, which yielded consistent results, while 1:5–1:30 dilutions were used for tobacco filler with low ammonia concentrations (Fig. 1). The reason for the low ammonia levels in the 1:5–1:30 dilutions was that the concentration of interfering substances in the ammonia extract was high. The effects of interfering substances were not observed when the extracts were prepared at a 1:50 dilution. The interfering substances in the ammonia extract did not affect ammonia analysis at dilutions of 50 times or more in this analysis method.

### Comparison between colorimetric method and ion chromatography

**Fig. 2:**
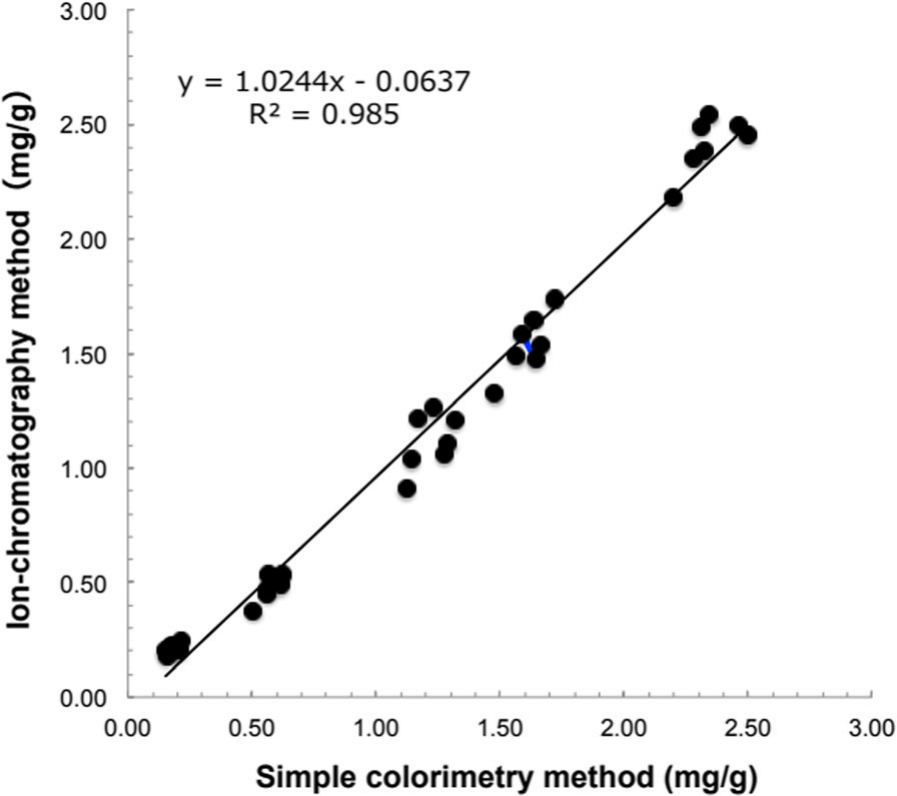
Comparison of the colorimetric method and ion chromatography for determining the ammonia levels in tobacco filler

The colorimetric method’s limit of quantification (LOQ) was 0.05 μg/mL (0.18 mg/g of tobacco filler). The linear region of the standard curve was 0.05–1.0 μg/mL, with a coefficient of determination 0.9991. This LOQ was higher than that of ion chromatography (0.003 μg/mL) [21]. However, the proposed method was applicable for the analysis of ammonia in tobacco filler. Our 3R4F data (1.14 ± 0.2 mg/g, *n* = 5) that were obtained using the colorimetric method and ion chromatography were comparable to those previously reported using ion chromatography [10, 16] (Table 1). Figure 2 shows the ammonia levels in the tobacco fillers extracted from five cigarette brands (3R4F, 1R5F, CM6, Marlboro Red, and Marlboro Gold) determined by the colorimetric method and ion chromatography. A linear relationship was observed between the ammonia levels determined by the two methods. The obtained coefficient of determination (*R*^2^) exceeded 0.985 (*y* = 1.0244*x* − 0.0637). These results demonstrate the applicability of the colorimetric method to the analysis of ammonia in tobacco filler.

### Determination of ammonia levels for Japanese cigarette brands

**Fig. 3:**
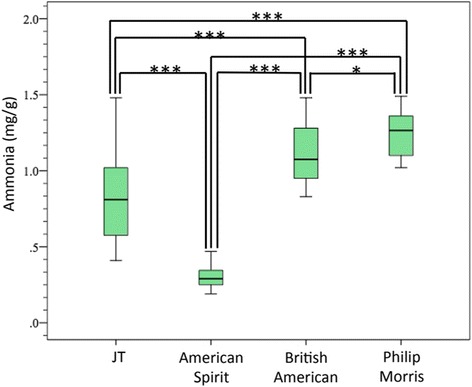
Ammonia levels in the tobacco filler of commercially marketed cigarettes in Japan (manufactured by Japan Tobacco Inc. (JT), Natural American Spirit, British American Tobacco, and Philip Morris Japan). The differences between the ammonia levels in cigarettes of different companies were analyzed by one-way ANOVA, followed by the Bonferroni post hoc test (*p < 0.05, ***p < 0.001)

Table 2 shows the ammonia levels in cigarette filler by cigarette brand. The mean ammonia level of all cigarette brands was 0.87 ± 0.40 mg/g, ranging from 0.22 mg/g for American Spirit Light to 1.45 mg/g for Marlboro Black Menthol Box. The coefficient of variation from the five measurements was 2.3–11.7% for each cigarette brand. The mean ammonia level of the Japanese Tobacco (JT) cigarette brands was significantly higher (0.83 ± 0.28 mg/g, *n* = 55) than that of Natural American Spirit brands (0.30 ± 0.08 mg/g, *n* = 40) and lower than those in the brands of the other two other companies (British American Tobacco (BAT) 1.11 ± 0.19 mg/g, *n* = 40; Philip Morris 1.24 ± 0.15 mg/g, *n* = 40) (*p* < 0.001 by Bonferroni test) (Fig. 3). Our results are consistent with those reported by Watson et al. [10], in that ammonia levels of the 34 American cigarette brands manufactured by three different tobacco companies differed significantly [10].

**Table 2:** Ammonia levels in tobacco filler extracted from commercially marketed cigarette brands in Japan (*n* = 5)

Cigarette brands	NH_3_ (mg/g) Mean ± SD
(a) Japan Tobacco Inc.	
MEVIUS ONE 100’s Box	0.77 ± 0.03
MEVIUS EXTRA LIGHTS	1.03 ± 0.03
MEVIUS SUPER LIGHTS	0.83 ± 0.06
MEVIUS LIGHTS	1.02 ± 0.04
MEVIUS ORIGINAL	0.86 ± 0.02
Seven Stars	0.45 ± 0.03
WAKABA	0.50 ± 0.02
echo	0.55 ± 0.04
CASTER One 100’s Box	1.04 ± 0.04
CASTER Mild	0.63 ± 0.04
CABIN MILD Box	1.40 ± 0.05
(b) NATURAL AMERICAN SPIRIT	
AS REGULAR Box	0.29 ± 0.02
AS LIGHT	0.22 ± 0.03
AS ORGANIC	0.34 ± 0.03
AS PERIQUE BOX	0.47 ± 0.01
AS ULTRA LIGHT	0.34 ± 0.02
AS MENTHOL LIGHT	0.24 ± 0.02
AS MENTHOL ULTRA LIGHT	0.27 ± 0.03
AS MENTHOL ONE	0.28 ± 0.03
(c) BRITISH AMERICAN TOBACCO	
KENT 1 100’s Box	1.38 ± 0.03
KENT iBoost 5 100’s Box	0.87 ± 0.04
KOOL MILDS Box	1.25 ± 0.07
KOOL NATURAL 8 Box	1.10 ± 0.03
CAPRI CHARCOAL SUPER SLIMS	0.89 ± 0.04
PALLMALL FK Box	1.38 ± 0.07
Lucky strike Box	1.02 ± 0.06
Lucky strike Light Box	1.01 ± 0.05
(d) PHILIP MORRIS	
Marlboro Box	1.07 ± 0.03
Marlboro gold ORIGINAL Box	1.05 ± 0.03
Marlboro LIGHTS MENTHOL Box	1.38 ± 0.03
Marlboro BLACK MENTHOL Box	1.45 ± 0.05
Marlboro ICE BLAST KS Box	1.32 ± 0.04
LARK MILDS KS Box	1.11 ± 0.06
LARK 100’s	1.25 ± 0.06
PARLIAMENT 100’s Box	1.30 ± 0.06

The average ammonia level in JT cigarette brands, which have high market shares in Japan, was lower than that of international brands (average of 1.14 ± 0.61 mg/g tobacco). These results were consistent with a report by the WHO [1]. The WHO reported that ammonia technology is a legacy technology. Currently, as there are 600 substance additives besides ammonia contained in tobacco products (including cocoa, caramel color, menthol, and rum and its flavors) [6]. These additives increase the alkalinity of cigarette smoke. Therefore, the contribution of ammonia to the alkalinity of cigarette smoke is considered to be low. The European Union and WHO have published a fact sheet for the regulation of additives based on articles 9 and 10 of the WHO Framework Convention on Tobacco Control (FCTC), which specify the regulation of the contents of tobacco products and regulation of tobacco product disclosures, respectively. However, the average ammonia level in JT brands was higher than that in domestic Canadian brands, which had an average of 0.26 ± 0.08 mg/g tobacco [9]. Initiatives to regulate tobacco products in Japan are behind those overseas. These results suggest that the ammonia levels in Japanese cigarette brands should be reduced further.

### Determination of ammonia levels in sidestream smoke

The colorimetric method was also used to analyze the ammonia levels in sidestream smoke. The ammonia levels were analyzed at the fishtail, CFP, and impinger stand of the sidestream smoking machine system. The fishtail and CFP collect particles from the sidestream smoke, and the impinger collects the gas phase. The ammonia levels in the sidestream smoke of 3R4F at the fishtail, CFP, and impinger stage were 0, 137, and 6312 μg/cigarette, respectively. The ammonia in sidestream smoke was mostly in gaseous phase and had an alkaline pH. We analyzed the ammonia levels in the sidestream smoke of cigarette brands that were found to contain high (CABIN and Marlboro Black Menthol) or low (American Spirit and Seven Stars) tobacco filler ammonia levels. The ammonia levels in the sidestream smoke of CABIN, Marlboro Black Menthol, American Spirit Light, and Seven Stars were 5.89 ± 0.28, 5.23 ± 0.12, 6.92 ± 0.56, and 4.14 ± 0.19 mg/cigarette, respectively (Table 3), and were all higher than the ammonia levels in their tobacco filler. Thus, the ammonia in sidestream smoke not only originates from tobacco filler but also from the burning of the cigarette. According to the WHO, the ammonia compounds detected in cigarette smoke are likely generated by the pyrolysis of nitrogenous compounds, such as amino acids and nicotine, and not directly transferred from filler to the smoke [1].

**Table 3:** Ammonia levels in sidestream smoke of four cigarette brands (*n* = 5)

Cigarette brands	Ammonia(mg/cigarette)
	Mean ± SD
CABIN Mild Box	5.89 ± 0.23
Marlboro BLACK MENTHOL Box	5.23 ± 0.12
American Spirit Light	6.92 ± 0.56
Seven Stars	4.14 ± 0.19

This study demonstrated the feasibility of the spectrophotometric method for the determination of ammonia levels in tobacco filler and sidestream smoke by using commercially available ammonia reaction reagents. The results of the analysis of the reference cigarettes by our method and ion chromatography were similar (i.e., WHO SOP). Diluting tobacco filler extracts by 50 times or more was required for analysis. In the future, this method will be tested for analyzing ammonia in mainstream smoke, smokeless tobacco, and new tobacco products, such as “IQOS” and “glo.”

## Conclusions

We developed a simple colorimetric method to measure the ammonia levels in tobacco filler and sidestream smoke using an absorption spectrometer and two reagents (sodium nitroprusside and sodium dichloroisocyanurate). A linear relationship was observed between the tobacco filler ammonia levels determined using our calorimetric method and those determined by ion chromatography. Our method could also be used to analyze ammonia in sidestream smoke. There were significant differences between the ammonia levels of the 35 commercially marketed cigarette brands in Japan manufactured by four tobacco manufacturers. Over 90% of the ammonia in sidestream smoke was in gaseous phase. In addition, ammonia levels were higher in the sidestream smoke than those in tobacco filler, suggesting that ammonia was produced as the cigarette burned.
